# Thyroid cancer risk prediction model using m6A RNA methylation regulators: integrated bioinformatics analysis and histological validation

**DOI:** 10.18632/aging.204525

**Published:** 2023-02-15

**Authors:** Wei Zhou, Junchao Lin, Jinqiang Liu, Rui Zhang, Aqiang Fan, Qibin Xie, Liu Hong, Daiming Fan

**Affiliations:** 1 Xijing Hospital of Digestive Diseases, The Fourth Military Medical University, Xi’an, Shaanxi 710032, China

**Keywords:** thyroid cancer, N6-methyladenosine (m6A), The Cancer Genome Atlas (TCGA), m6Acluster, geneCluster, m6Ascore

## Abstract

Background: Epigenetic reprogramming has been reported to play a critical role in the progression of thyroid cancer. RNA methylation accounts for more than 60% of all RNA modifications, and N6-methyladenosine (m6A) is the most common modification of RNAs in higher organisms. The purpose of this study was to explore the related modification mode of m6A regulators construction and its evaluation on the clinical prognosis and therapeutic effect of thyroid cancer.

Methods: The levels of 23 m6A regulators in The Cancer Genome Atlas (TCGA) were analyzed. Differentially expressed genes (DEGs) and survival analysis were performed based on TCGA-THCA clinicopathological and follow-up information, and the mRNA levels of representative genes were verified using clinical thyroid cancer data. In order to detect the effects of m6A regulators and their DEGs, consensus cluster analysis was carried out, and the expression of different m6A scores in Tumor Mutation Burden (TMB) and immune double antibodies (PD-1 antibody and CTLA4 antibody) were evaluated to predict the correlation between m6A score and thyroid cancer tumor immunotherapy response.

Results: Different expression patterns of m6A regulatory factors were detected in thyroid cancer tumors and normal tissues, and several prognoses related m6A genes were obtained. Two different m6A modification patterns were determined by consensus cluster analysis. Two different subgroups were established by screening overlapping DEGs between two m6A clusters, with cluster A having the best prognosis. According to the m6A score extracted from DEGs, thyroid cancer patients can be divided into high and low score subgroups. Patients with lower m6A score have longer survival time and better clinical features. The relationship between m6A score and Tumor Mutation Burden (TMB) and its correlation with the expression of PD-1 antibody and CTLA4 antibody proved that m6A score could be used as a potential predictor of the efficacy of immunotherapy in thyroid cancer patients.

Conclusions: We screened DEGs from cluster m6A and constructed a highly predictive model with prognostic value by dividing TCGA-THCA into two different clusters and performing m6A score analysis. This study will help clarify the overall impact of m6A modification patterns on thyroid cancer progression and formulate more effective immunotherapy strategies.

## INTRODUCTION

Thyroid cancer is the most common malignant tumor of the endocrine system, ranking ninth in the incidence of malignant tumors worldwide. Its incidence has increased significantly in recent years, with more than 580,000 new cases worldwide in 2020 [1]. According to the pathological classification, thyroid cancer can be divided into differentiated, myeloid, poorly differentiated, and undifferentiated types. Differentiated thyroid cancer accounts for more than 90% of thyroid cancer cases and can be further divided into papillary thyroid cancer (PTC, approximately 80–85%), follicular thyroid cancer (approximately 10–15%), anaplastic thyroid cancer (<2%), and poorly differentiated thyroid cancer (<2%) [2]. Environmental and genetic factors play important roles in the pathogenesis of thyroid cancer [3], and genetic changes, such as BRAF and RAS mutations or RET/PTC rearrangement leading to activation of the oncogenic MAPK pathway, are closely related to the occurrence of thyroid cancer [4]. Therefore, it is important to analyze the pathogenesis of this disease at the molecular level.

An increasing number of studies have shown that post-transcriptional RNA modifications play an important role in the occurrence and development of various malignant tumors [5–7]. In the context of tumor progression, RNA and histone changes at epigenetic and genetic levels have been widely studied, and many therapeutic methods have been developed [8–10]. N6-methyladenine (m6A), a type of RNA modification, refers to methylation at the N6 position of the adenine base, and it has the highest endogenous abundance and widely exists in eukaryotic RNAs [11–13]. In terms of molecular mechanisms, m6A participates in almost all processes of RNA metabolism, including RNA transport, splicing, translation, and degradation, and plays a vital role in regulating gene expression [14–16]. By regulating the expression of tumor-related genes, m6A participates in the regulation of tumor proliferation, invasion, and metastasis, and its regulation is dynamic and reversible in various pathophysiological processes [17–19].

Immunotherapy can improve patient prognosis by enhancing antitumor immunity [20]. Immune checkpoint inhibitors (ICIs) that target T cells include anti-programmed death protein 1 (PD-1), anti-programmed death ligand 1 (PD-L1) antibodies, and anti-cytotoxic T lymphocyte-associated protein 4 (CTLA4) [21, 22]. Studies on immunogenic tumors, such as melanoma, Hodgkin’s lymphoma, and non-small cell lung cancer, have confirmed that the combination of ICIs and other kinds of antitumor drugs could expand the benefits to patients [23, 24]. Researchers are constantly exploring the clinical practice of introducing PD-1/PD-L1 as a biomarker and treatment strategy for thyroid cancer [25]. However, not all patients respond well to ICI treatment [26]. Therefore, the optimization of detection methods and selection of treatment regimens are still important challenges faced by clinicians [27].

In this study, we integrated transcriptomic and genomic data of 506 thyroid cancer samples from The Cancer Genome Atlas (TCGA) database to evaluate m6A modification patterns and comprehensively assess its relationship with the infiltration characteristics of tumor microenvironment (TME) cells. Through non-negative matrix factorization clustering, three different m6A modification patterns were identified, and three groups of different genes were screened according to different m6A genotypes. A quantitative “m6A score” system was further established to define different m6A modification patterns. The findings suggest that m6A plays an integral role in the formation of the tumor microenvironment and may serve an important function in the treatment and prognosis of thyroid cancer.

## RESULTS

### Genetic variation of m6A regulators in thyroid cancer

**Figure 1:**
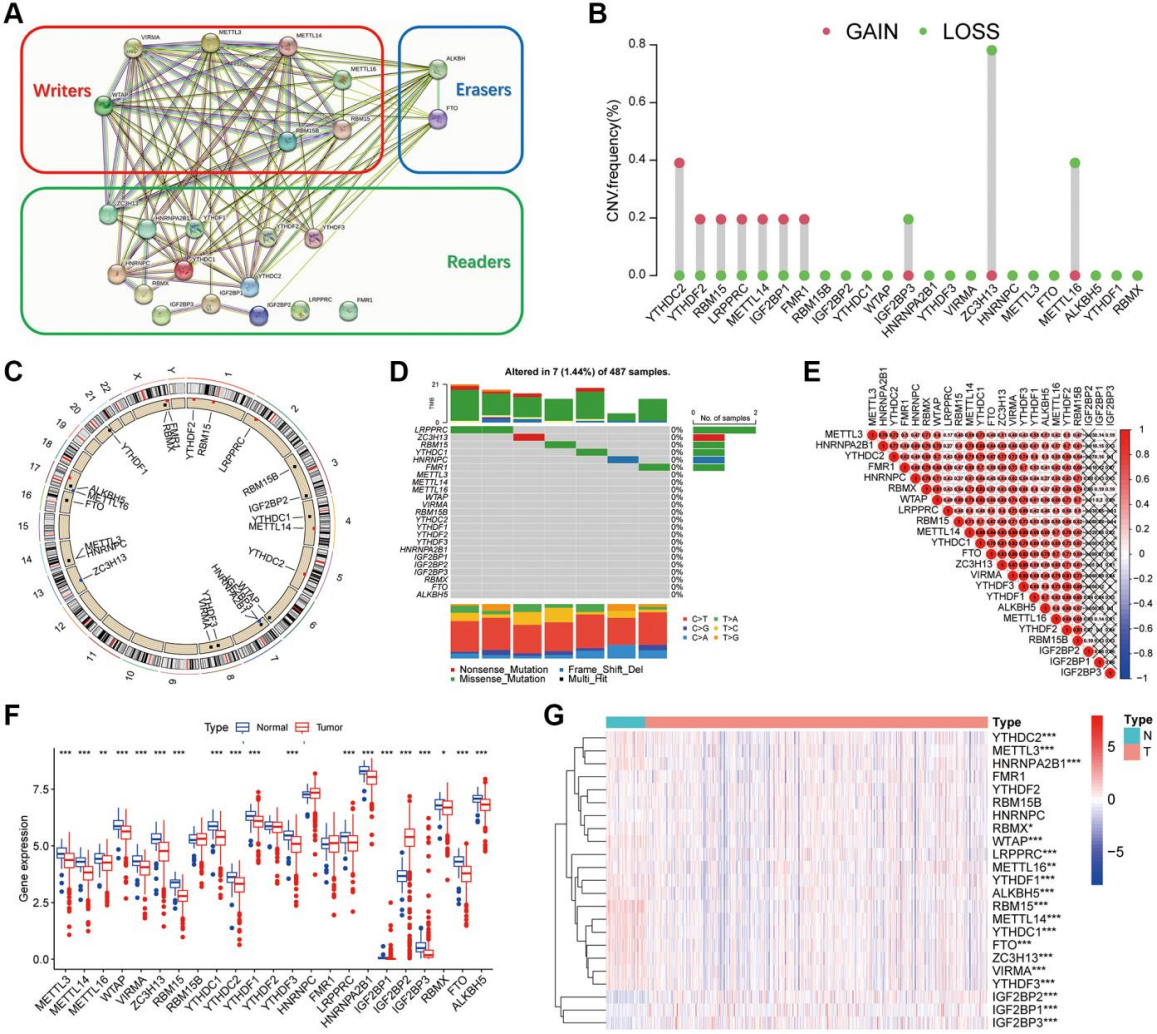
**Genetic variation of m6A regulators in thyroid cancer.** (**A**) The protein-protein interaction (PPI) network of 23 m6A regulators. (**B**) The mutation frequency. (**C**) The location of the change of m6A regulator CNV on chromosome. (**D**) m6A waterfall plot. The right vertical coordinate represents m6A regulators, and the left vertical coordinate represents the mutation rate of m6A regulators in thyroid cancer. (**E**) Pearson correlation analysis shows the correlation of 23 m6A methylation modification regulators in thyroid cancer. (**F**) m6A methylation regulators expression in thyroid cancer. The figure shows the expression of 23 m6A regulators in thyroid cancer tumors and normal specimens. (**G**) The difference of mRNA expression levels of 23 m6A regulators between normal and thyroid cancer samples. The asterisk indicates statistical *p* value (^*^P < 0.05, ^**^ *P* < 0.01, ^***^P < 0.001).

Figure 1A showed the protein-protein interaction (PPI) network of 23 m6A regulators, including eight writers, 13 readers, and two erasers. Different colors used to distinguish the functions of the regulators using the STRING database. Further analysis (Figure 1B) revealed CNV mutations in m6A regulators. YTHDC2, YTHDF2, RBM15, LRPPRC, METTL14 and FMR1 showed CNV frequency gain, in contrast, IGF2BP2, ZC3H13 and METTL16 showed CNV frequency loss. The CNV changes of 23 m6A regulators on chromosomes were shown in Figure 1C. As shown in Figure 1D mutation frequency analysis, 6 m6A related genes were mutated among 7 of 487 samples (1.44%). Figure 1E showed the expression of 23 m6A methylation regulators in the thyroid cancer tissues and normal tissues. In the heatmap, red indicated high expression and blue indicated low expression. It was observed that 23 m6A regulators had different expression characteristics in tumor and normal tissues. To determine whether these genetic variations affect the expression of m6A regulators in patients with thyroid cancer, we studied the mRNA expression levels of regulators in tumor and normal samples. The differential expression of m6A gene in thyroid cancer was analyzed using TCGA database. In block diagram Figure 1F, red represented thyroid cancer tissue and blue represented normal tissue. It could be seen from the figure that most m6A genes differentially expressed in cancer and normal tissues. Pearson correlation analysis showed the correlation among the 23 m6A genes. In Figure 1G, red and blue indicated the positive and negative correlations, respectively. As shown in the figure, VIRMA was highly correlated with YTHDF3, METTL14, and ZC3H13, with correlation coefficients of 0.92, 0.89, and 0.87, respectively. YTHDC1 was also highly correlated with METTL14 with a correlation coefficient of 0.89. Table 1 showed the expression level of 23 m6A regulators in thyroid cancer tissues and normal tissues.

**Table 1:** The expression levels of 23 m6A regulators in TCGA-THCA and normal tissues.

**Gene**	**Normal (median)**	**Tumor (median)**	**logFC**	***p*-value**	***P* symbol**
IGF2BP2	11.94091936	40.50527386	1.762195844	8.41E-24	^***^
RBM15	9.226988352	6.034930992	−0.612522561	4.87E-22	^***^
YTHDC1	57.38977318	41.05247029	−0.483324635	2.95E-15	^***^
METTL14	18.33430627	13.11828989	−0.482966017	8.53E-15	^***^
FTO	18.36648365	13.08475892	−0.489188098	1.49E-12	^***^
ZC3H13	37.88995144	27.22970004	−0.476634201	5.57E-11	^***^
IGF2BP3	0.490382364	0.892929291	0.864638841	6.10E-11	^***^
HNRNPA2B1	320.5051508	261.1527411	−0.295453696	6.37E-10	^***^
WTAP	59.99919573	47.90170005	−0.324866303	5.25E-09	^***^
ALKBH5	134.8081736	113.6898924	−0.245803975	6.41E-08	^***^
IGF2BP1	0.048973371	0.047495885	−0.044194993	6.42E-08	^***^
YTHDC2	11.11029344	8.93724255	−0.313995238	4.15E-07	^***^
YTHDF3	42.34521438	34.01845178	−0.31588145	4.54E-07	^***^
KIAA1429	19.19407034	15.63908256	−0.295504803	5.78E-07	^***^
METTL3	24.46770127	20.21351981	−0.275558005	2.83E-06	^***^
YTHDF1	78.59670676	67.79570638	−0.213274956	4.28E-06	^***^
LRPPRC	40.77588123	35.37785033	−0.204869668	0.000151512	^***^
METTL16	20.69897985	18.64734899	−0.150589123	0.0046163	^**^
RBMX	112.698666	102.968029	−0.13027398	0.014140605	^*^
YTHDF2	58.75080248	55.69420552	−0.077081322	0.088825874	ns
HNRNPC	153.0058443	159.6669495	0.06147895	0.101208289	ns
FMR1	33.60589753	34.29579162	0.029317122	0.798681961	ns
RBM15B	38.27544113	38.53053764	0.009583312	0.825583473	ns

ns *P* > 0.05; ^*^P < 0.05; ^**^P < 0.01; ^***^P < 0.001.

### Relationship between m6A related genes and prognosis of thyroid cancer

**Figure 2:**
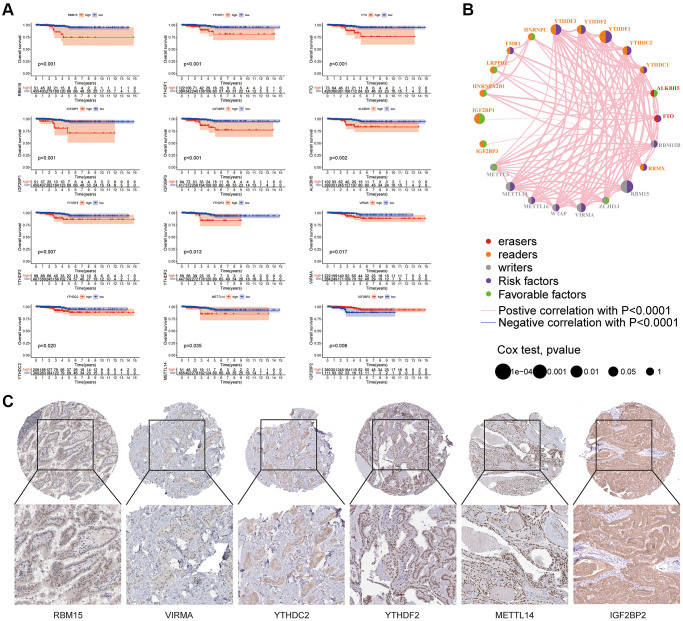
**Relationship between m6A related genes and prognosis of thyroid cancer.** (**A**) In the survival curve, the abscissa is the survival time (years) and the ordinate is the survival rate. (**B**) The m6A prognosis network shows the expression and interaction of 23 m6A regulators in thyroid cancer. (**C**) Human protein Atlas (https://www.proteinatlas.org/) is used to analyze the protein expression of some m6A molecules related to prognosis in thyroid cancer tissue.

As shown in Figure 2A, TCGA database was used for m6A survival analysis, and nine m6A genes related to the prognosis of thyroid cancer patients were screened. In Table 2, HR was the risk value, HR>1 means the risk increases, HR<1 means the risk decreases, and HR.95L and HR.95h were the risk fluctuation ranges. According to the relationship between m6A related genes and prognosis, Figure 2B showed the prognosis network, which reflects the comprehensive situation of m6A regulator interaction, regulator connection, and prognostic significance for thyroid cancer patients. We found that not only m6A regulators in the same functional category showed a significant correlation in expression, but also among writers, readers, and erasers. As shown in Figure 2C, the protein expression of m6A molecules related to prognosis in thyroid cancer tissues was analyzed using a human protein map (https://www.proteinatlas.org/). Immunohistochemical images showed the expression levels of RBM15, VIRMA, YTHDC2, YTHDF2, METTL14, and IGF2BP2 proteins in tumor tissues.

**Table 2:** Survival analysis of 23 m6A regulators in thyroid cancer.

**Gene**	**HR**	**HR.95L**	**HR.95H**	***p*-value**	***P* symbol**
IGF2BP1	1.550281322	0.829728968	2.896574989	1.87E-05	^***^
IGF2BP3	1.024058896	0.978769378	1.071444047	3.23E-05	^***^
FTO	1.093703115	0.990086791	1.208163279	0.000963146	^***^
METTL14	1.056388222	0.934489434	1.194188009	0.0013232	^**^
ALKBH5	1.012323505	0.998468838	1.026370418	0.001987976	^**^
RBM15	1.355310563	1.028093504	1.786672822	0.004829401	^**^
IGF2BP2	0.977475317	0.952836017	1.002751763	0.008285184	^**^
YTHDF1	1.018635523	0.998828253	1.038835581	0.009836063	^**^
YTHDF3	1.038398012	1.000004621	1.078265448	0.010888568	^*^
YTHDF2	1.01763239	0.981140235	1.05548182	0.018658739	^*^
YTHDC2	1.157047747	0.9788176	1.367731321	0.024447184	^*^
VIRMA	1.085846511	0.982094849	1.200558831	0.030185204	^*^
FMR1	0.986686411	0.948506148	1.026403547	0.062856084	ns
LRPPRC	1.014983114	0.984097928	1.046837609	0.079568495	ns
METTL3	1.003110125	0.933186276	1.078273384	0.090903573	ns
HNRNPC	0.997300779	0.985766742	1.008969771	0.094004403	ns
ZC3H13	0.995183256	0.952081924	1.040235812	0.10464568	ns
RBMX	0.99999406	0.983459992	1.016806101	0.108771912	ns
YTHDC1	1.009506738	0.971320357	1.049194373	0.119336858	ns
METTL16	1.01375848	0.936714379	1.097139404	0.130851476	ns
HNRNPA2B1	1.000157356	0.99294754	1.007419524	0.131226368	ns
WTAP	1.005355791	0.96790777	1.044252664	0.134656417	ns
RBM15B	1.009332968	0.964093249	1.056695543	0.140563803	ns

ns *P* > 0.05; ^*^P < 0.05; ^**^P < 0.01; ^***^P < 0.001.

### Determination of m6A modification mode

**Figure 3:**
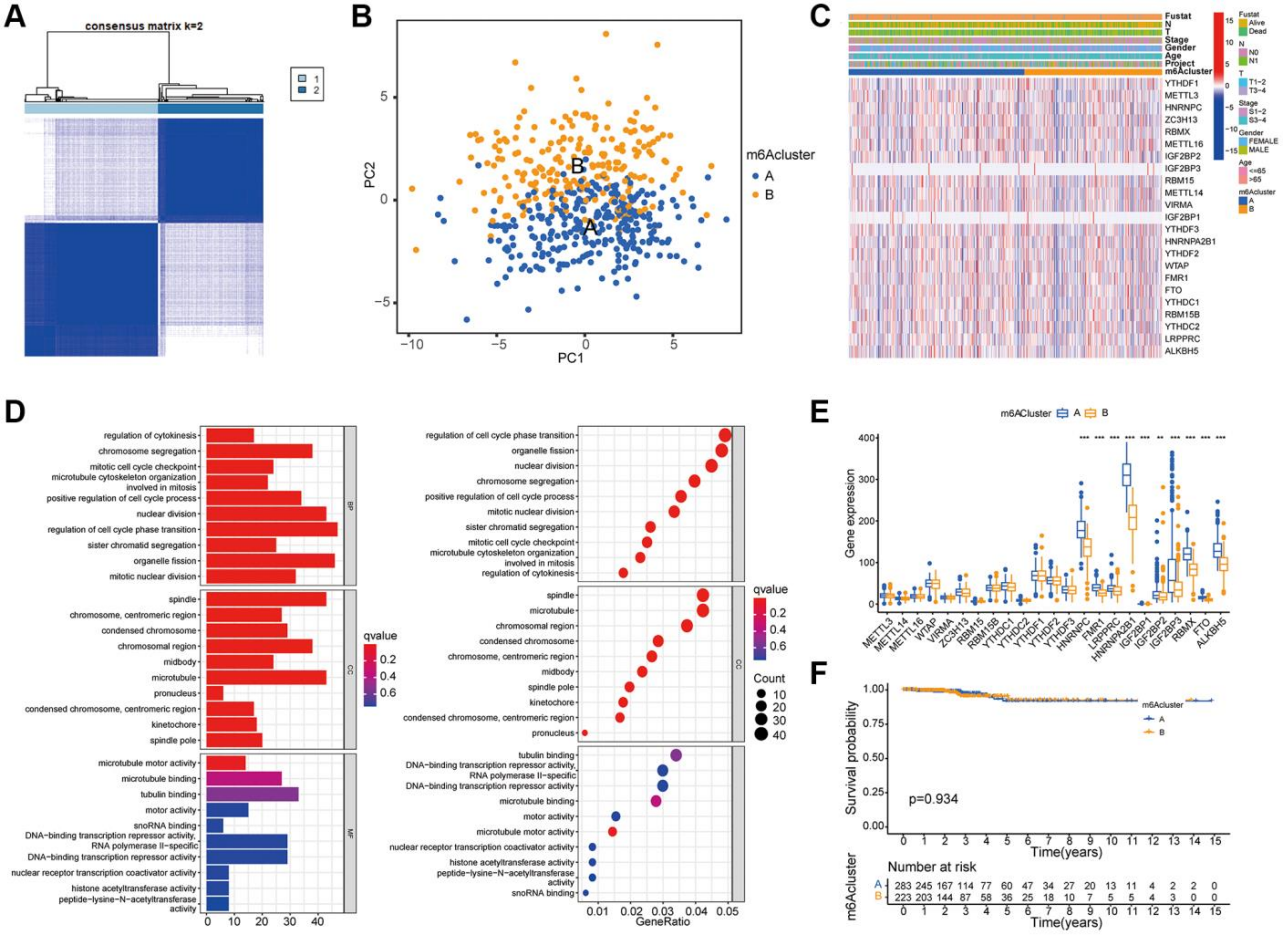
**Determination of m6A modification mode.** (**A**) According to the expression similarity of m6A RNA methylation regulator, 506 thyroid cancer patients in TCGA cohort were divided into m6A Cluster A and B. (**B**) PCA analysis shows that m6A related genes can distinguish the two groups of m6A genotyped samples. (**C**) The Heatmap shows an unsupervised cluster of 23 m6A regulators in TCGA-THCA. (**D**) GO enrichment analysis was performed on the difference genes screened by comparison between the two groups of m6A cluster to observe the functions of these genes. The ordinates of the histogram and bubble diagram represent the name of GO, which can be divided into three categories: BP (biological process), CC (Cell Component), and MF (Molecular function). (**E**) The expression of 23 m6A regulators in the two groups of m6A cluster. The asterisk indicates statistical *P* value (^*^P < 0.05; ^**^P < 0.01; ^***^P < 0.001). (**F**) Survival analysis for RFS among two m6Aclusters. Kaplan–Meier curves and log-rank *P* values are shown in the graph, and the numbers at risk are shown at the bottom.

The category discovery tool “sense clusterplus” was used to uniformly cluster the data of patients with thyroid cancer based on the m6A methylation regulator. Figure 3A and Supplementary Figure 1A–1J showed that, between *k* = 2 and 9, the most stable clustering results can be obtained when *k* = 2. PCA (Figure 3B) showed that this classification method could effectively distinguish samples. In Figure 3C, thermographic analysis was used to display the distribution of different clinical features in the two m6A grouped samples. In Figure 3D, histograms and bubble diagrams were drawn using GO enrichment analysis to observe the functional types, mainly involving differential genes. It could be seen from the figure that the functions mainly focus on cell cycle regulation and cell mitosis. In the block diagram Figure 3E, the abscissa represented the m6A related gene, and the ordinate represents the gene expression level. Some m6A molecules in the figure are differentially expressed in genotype, among which m6A regulator is highly expressed in genotype A. Figure 3F showed that Kaplan Meier survival curve based on m6A grouping has no significant difference among groups (*P* = 0.934).

### Construction of m6A gene subgroup

**Figure 4:**
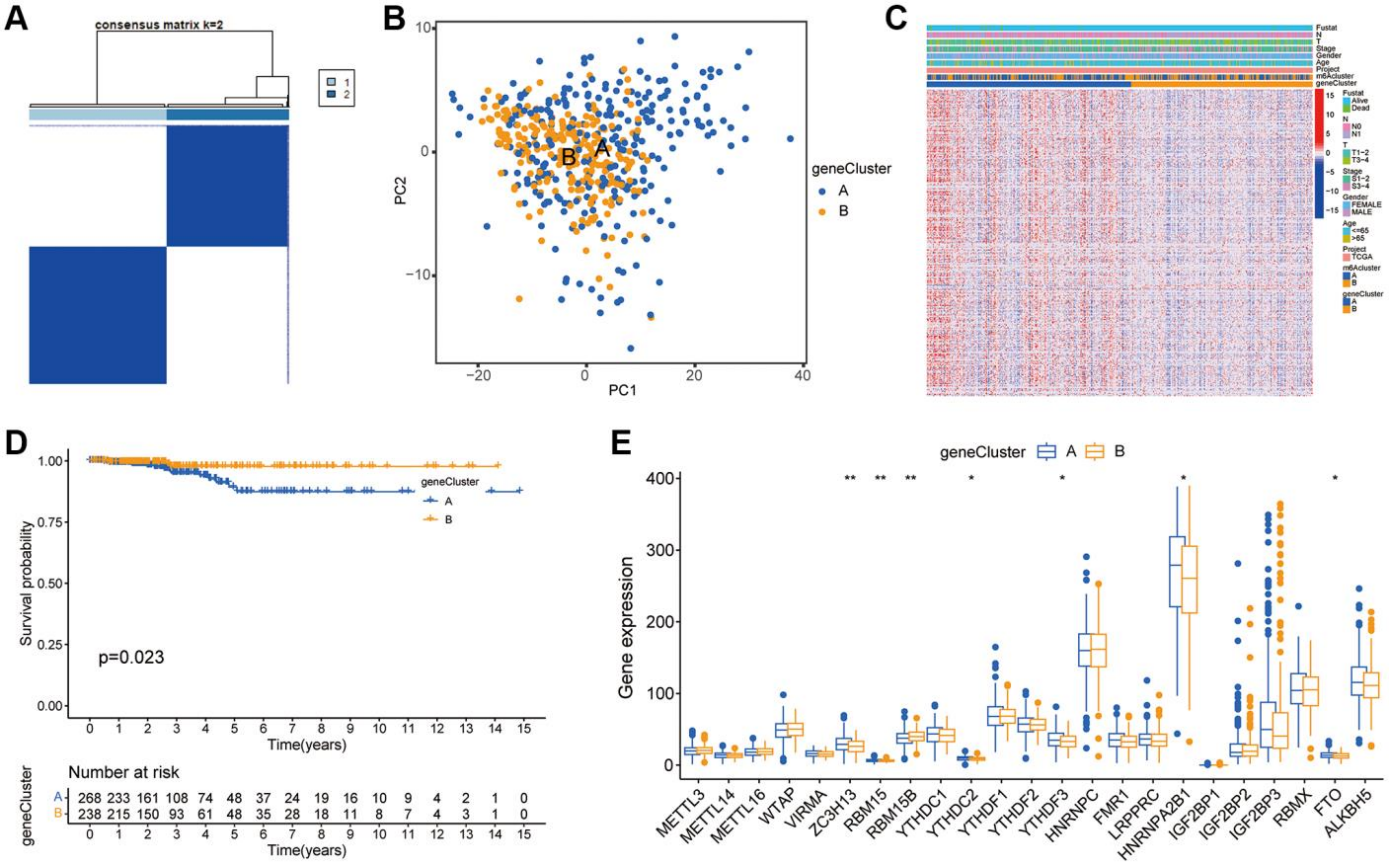
**Construction of m6A gene subgroup.** (**A**) By screening the overlapping DEGs between two m6A clusters and conducting unsupervised consensus cluster analysis, the samples are classified into two types according to the internal correlation. The types 1 and 2 correspond to gene cluster-A and gene cluster-B respectively. (**B**) PCA analysis shows that m6A related DEGs can distinguish two groups of m6A cluster samples. (**C**) Heatmap is drawn for m6A cluster of the two groups according to different types. The abscissa in the figure represents samples and the ordinate represents m6A related genes. (**D**) Kaplan-Meier curve is used to evaluate the survival of phenotypic m6A related gene characteristics, and the results show that the prognosis of genotype A is significantly better than that of genotype B (*P* = 0.023). (**E**) Expression of 23 m6A regulators in three gene clusters. The top and bottom of the box represent the quartile range of values, the lines in the box represent the median, and the colored dots represent outliers. The asterisk indicates the statistical *p* value (^*^P < 0.05; ^**^P < 0.01; ^***^P < 0.001).

Although the consistent clustering algorithm based on m6A regulator expression divided thyroid cancer patients into two m6A modified phenotypic patterns, the potential genetic changes and expression barriers of these phenotypes were still unclear. Therefore, we applied the empirical Bayesian method to screen overlapping differentially expressed genes (DEGs) between two m6A clusters and conducted unsupervised consistent cluster analysis to obtain two different m6A gene feature subsets, which were defined as gene clusters A and B (Figure 4A and Supplementary Figure 2A–2J). Figure 4B showed the PCA results. A heatmap Figure 4C was drawn according to the genotype, in which the abscissa represented the sample, the ordinate represents the gene, and blue and yellow represent the genome. The number of patients with clinical characteristics was higher in this group. As could be seen from the figure, the two component types had different expressions. Figure 4D showed the survival curves of patients with thyroid cancer. The results showed a significant difference between the two subtypes of m6A gene clusters A and B (*P* = 0.023). In the block diagram Figure 4E, the abscissa represented the m6A related gene, and the ordinate represents the gene expression level. It could be seen from the figure that some m6A molecules were differentially expressed between genotypes.

### Construction of m6A score system

**Figure 5:**
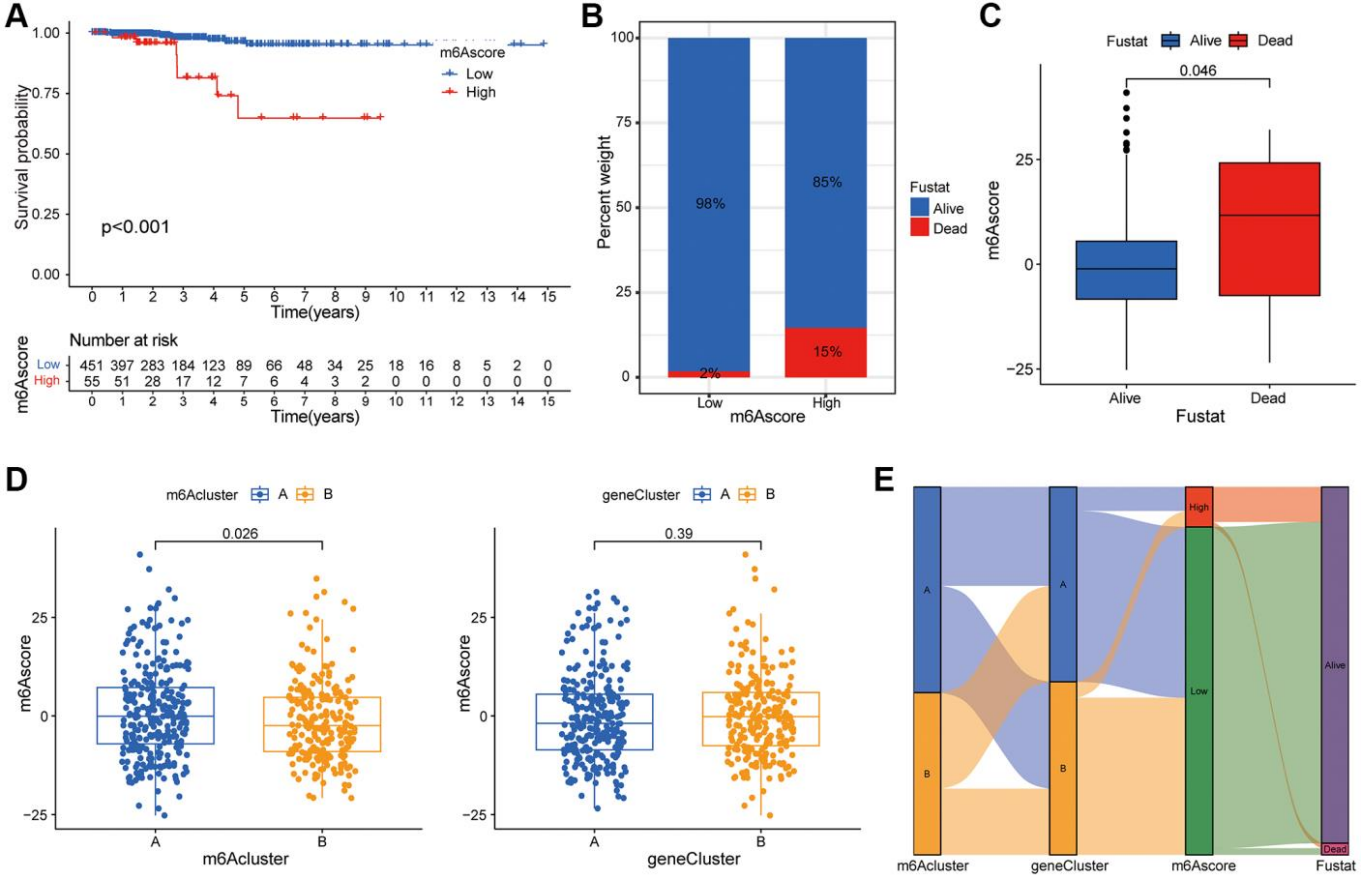
**Construction of m6A score system.** (**A**) Survival curve shows that the prognosis of thyroid cancer patients in m6A low rating group is significantly better than that in high rating group (*P* < 0.01). (**B**) Histogram shows the proportion of patients who survived or died within 5 years in the low or high m6A group. Comparison of survival and death: 98% and 2% in the low m6A score group, and 86% and 15% in the high m6A score group, respectively. (**C**) The abscissa in the boxplot represents the survival and death groups, and the ordinate is the m6A score. It can be seen that the m6A score in the death group is significantly higher than that in the survival group (*P* = 0.046). (**D**) There is a significant difference in m6A score between m6A cluster A and B (*P* = 0.026), while m6A score shows no significant difference between genotypes (*P* = 0.39). (**E**) Alluvial diagram is drawn based on m6A cluster, genotype, m6A score and patient survival status, which shows the distribution of different genotypes.

PCA was used to obtain the m6A score of each sample based on the prognosis gene, and the best cut-off value was selected to divide the patients into high and low scores and to draw the survival curve. As shown in Figure 5A, there was a significant difference between the high and low scores of m6A, indicating that the prognosis of patients with low m6A was relatively good. The abscissa of histogram Figure 5B was m6A high score group and low score group, and the ordinate was the percentage of survival status within 5 years. As shown in the figure, the survival rate of the patients in the low group was relatively high. On the basis of the 5-year survival rate, patients with thyroid cancer were divided into survival and death groups. The m6A scores of the two groups of patients were compared. Block diagram Figure 5C showed that the m6A scores of deceased patients were higher than those of survivors. In Figure 5D, it could be seen that there was a significant difference in m6A scores between the two groups of m6A subtypes, with the highest score in m6A subtype a. There was no significant difference in m6A scores between the genotypes. Figure 5E was constructed based on m6A clustering, genotype, m6A score and survival status. The figure showed the distribution of different genotypes among the other genotypes.

### m6A score predicts the benefits of immunotherapy

**Figure 6:**
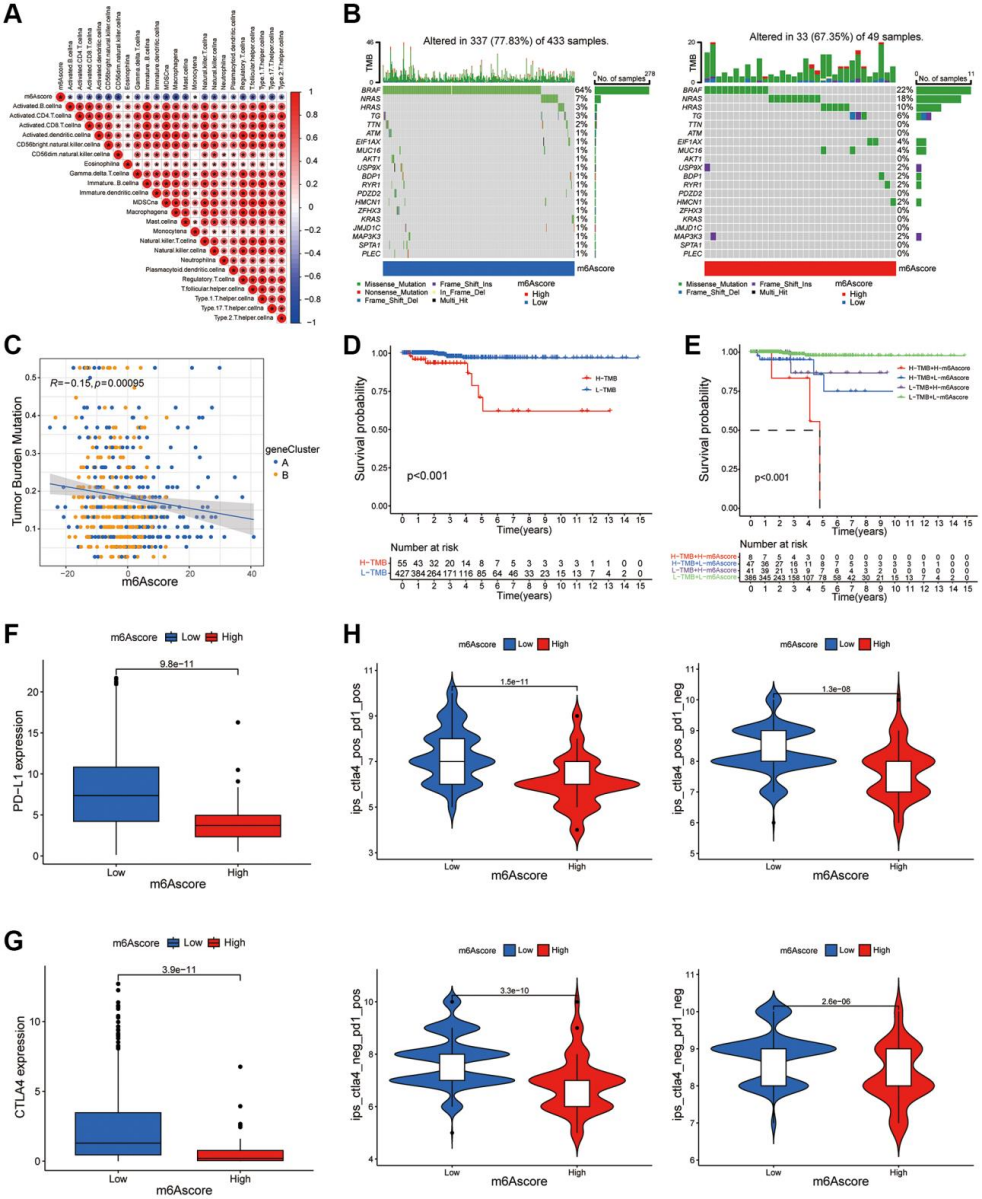
**m6A score predicts the benefits of immunotherapy.** (**A**) The correlation between m6A score and immune cells can be observed by immune correlation analysis. (**B**) In the waterfall plot, the abscissa is the sample, the ordinate is the mutation related gene, different colors represent different mutation types, and different base changes are shown below the graph. (**C**) Correlation analysis of m6Ascore and TMB value in thyroid cancer was performed through Spearman correlation analysis. (**D**) The survival curve shows that patients with low TMB had significantly better survival than those with high TMB (*P* < 0.001). (**E**) TMB and m6A score were compared in the survival curve, and the results shows that the survival rate of patients with low TMB and low m6A score is significantly higher than that of patients with high TMB and high m6A score (*P* < 0.001). (**F**) Box plot of PD-L1 expression in the low and high m6Ascore groups. The *P* value is shown in box plot. (**G**) Box plot of CTLA4 expression in the low and high m6Ascore groups. The *P* value is shown in box plot. (**H**) The expression levels of CTLA4 antibody and PD-1 antibody in high m6A score group and low m6A score group were compared.

Figure 6A showed that the m6A score was negatively correlated with most immune cells. According to the m6A score, two waterfall figures (Figure 6B) were drawn. Red indicates that the m6A score was high and blue indicates that the m6A score was low. By comparing the two figures, it could be seen that the mutation frequency of each gene in the high and low-rating groups was usually different, and the mutation frequency of the thyroid cancer tumor marker BRAF gene was higher in the low-rating group. Figure 6C showed a significance test between the m6A score and TMB to illustrate the relationship between TMB and m6A scores in patients with thyroid cancer. Correlation analysis showed a significant negative correlation between m6A score and TMB (R = −0.15, *P* = 0.00095). We conducted a survival analysis of the tumor mutation load. The results in Figure 6D showed that the survival rate of patients with low TMB was significantly higher than that of those with high TMB. Combined with TMB and m6A scores, the results in Figure 6E showed that the survival rate of patients with low TMB and low m6A scores was significantly higher than that of patients with high TMB and high m6A scores. To detect difference in PD-L1 expression in m6A score and support related immunotherapy, we detected the expression of PD-L1 and CTLA-4 in different m6A score groups. The results showed that the expression of PD-L1 and CTLA-4 in the low m6A score group was significantly higher than that in the high score group (Figure 6F, 6G). In Figure 6H, the abscissa was the m6A score and the ordinate was the immunotherapy score. Different expression levels of CTLA4 and PD-1 antibodies were detected in the positive (POS) and negative (NEG) groups. It could be seen that the immunotherapy scores of the low m6A scoring group were generally higher, indicating that the low m6A scoring group was superior to the high m6A scoring group in terms of immunotherapy benefits (*P* < 0.05).

### Clinical evaluation of m6A score

**Figure 7:**
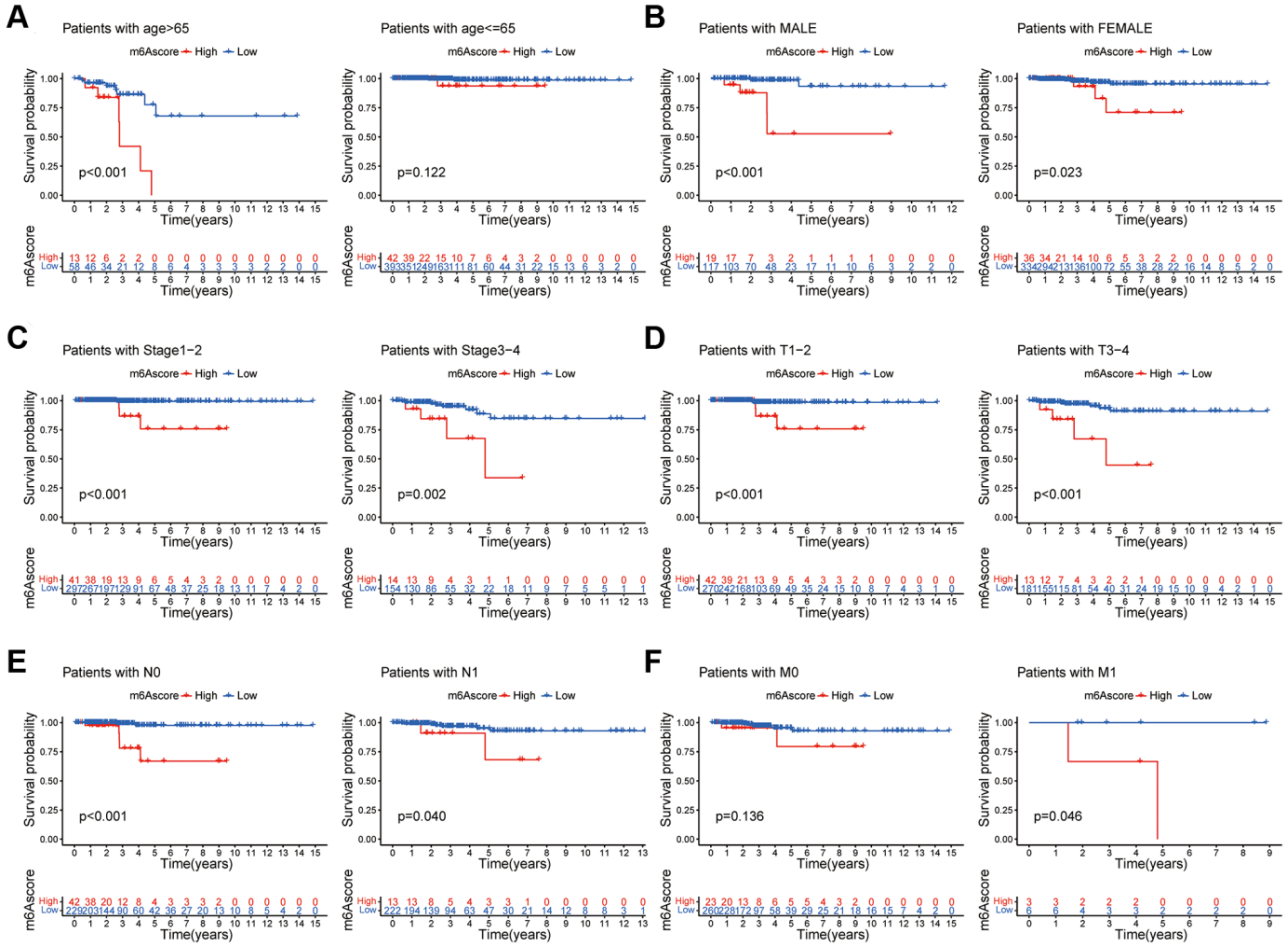
**Clinical evaluation of m6A score.** Survival analysis of different m6Ascore groups among thyroid cancer patients with Age (**A**), Gender (**B**), tumor Stage (**C**), T stage (**D**), N stage (**E**), and M stage (**F**).

The relationship between the m6A scores and clinical characteristics was shown in Figure 7. Figure 7A showed that among patients older than 65 years, the prognosis of patients with low m6A score was better than that of patients with high score. Figure 7B showed that, in both male and female patients, the prognosis of patients with low m6A scores was better than that of patients with high scores. Figure 7C showed that among patients with different tumor stages, the prognosis of patients with low m6A scores was better than that of patients with high score. Figure 7D showed that regardless of the T stage, the prognosis of patients with low m6A scores was better than that of patients with high scores. Figure 7E showed that the prognosis of patients with low m6A scores was better than that of patients with high scores, regardless of N stage. Figure 7E showed that the prognosis of patients with low m6A scores was better than that of patients with high score in the M1 stage.

## DISCUSSION

In this study, we comprehensively evaluated the m6A modification mode of thyroid cancer in TCGA database, constructed a gene cluster model based on the differentially expressed genes (DEGs) screened by m6A typing, and built a quantitative m6A modification mode scoring system through m6A typing.

To explore the prognostic biomarkers of thyroid cancer, it is necessary to elucidate the molecular mechanisms of thyroid cancer progression. Epigenetic reprogramming has been reported to be the key to tumor progression [28–30]; however, the role of mRNA post-transcriptional modification in thyroid cancer remains unclear. In recent years, N6 methyladenine (m6A) modification, a new type of RNA methylation, has become a research hotspot [31–36]. m6A modification refers to methylation modification at the N6 position of the adenine base. It is a type of RNA modification with the highest endogenous abundance and is involved in almost all RNA metabolic processes, including RNA transport, splicing, translation, and degradation. The level of m6A modification is dynamically regulated by the methylation enzyme coder (Writer) and demethylase eraser (Eraser), and the methylation modification information is read by combining it with the binding protein reader, as well as the fine regulation of downstream RNA transcription and translation processes. By regulating the expression of tumor-related genes, m6A plays a significant role in the processes of tumor development, such as proliferation, invasion, and metastasis [37]. Various m6A regulators may form complex network structures and interact to affect tumor progression [38]. However, the role of m6A modification and its regulators in the malignant progression of thyroid cancer remains unclear.

As epigenetic regulators, m6A regulators mainly function in the post-transcriptional modification of target genes. To analyze the downstream genes of the m6A regulator, we focused on the DEGs in the intersection set of the two thyroid cancer subgroups of m6A and screened prognostic risk genes using univariate Cox regression analysis. Based on these genes, consensus clustering analysis was performed to build a risk prediction model. The prognosis analysis clearly showed that the prognosis of patients in group B was significantly better than that of patients in group A, indicating the accuracy of the risk-prediction model for prognosis judgment.

Recent studies have revealed the interaction between TME immune cell infiltration and m6A modification, which cannot be fully explained by the RNA degradation mechanism [39–43]. Lan et al. found that, compared with oxaliplatin (OX)-sensitive patients, OX-resistant patients had more intensive macrophage infiltration in colorectal cancer tissues. Similarly, the total m6A RNA content and expression of METTL3, a key methyltransferase, increased in colorectal cancer tissues of OX-resistant patients. In addition, M2-polarized tumor-associated macrophages can induce OX resistance by improving METTL3-mediated m6A modification [44]. An increasing number of studies have shown that m6A regulatory factors play important roles in inflammation, tumor immunity, and antitumor therapy. However, the relationship between m6A methylation regulators and various clinicopathological features of thyroid cancer is not completely clear [45]. In addition, tumorigenesis is often characterized by the interaction of multiple tumor regulators in a highly coordinated manner to promote tumor progression. Owing to the limitations of conditions and technologies, previous studies have mainly focused on a single m6A regulator or a single immune cell type, and there are only a few studies on multiple m6A regulators that simultaneously mediate tumor development and TME invasion characteristics. Therefore, a comprehensive investigation of the infiltration characteristics of TME cells mediated by multiple m6A regulators will deepen our understanding of cancer immune regulation [46]. At present, accumulated bioinformatics data provide rich resources for the comprehensive analysis of m6A regulatory factors and TME immune regulation [47–49]. The use of bioinformatics analysis to identify different m6A decoration patterns in tumor and TME infiltrating cells is crucial to determine their role in antitumor immunity and to guide the formulation of antitumor immunotherapy strategies [50].

In order to further quantitatively explain the characteristics of m6A, we established a quantitative scoring system called “m6A score” to define different m6A modification modes, which will also provide a more accurate guide for immunotherapeutic strategies. We calculated the m6A score based on the expression of prognosis-related risk genes in the tumor dataset and divided the scores into high- and low-scoring groups according to the survival data. The high m6A score group was found to be significantly related to poor prognosis in thyroid cancer. In addition, the m6A score was negatively correlated with 23 immune-related cells. Therefore, the potential mechanism through which m6A methylation promotes thyroid cancer progression may play a role in regulating immune cell infiltration. In this study, we found that the m6A score was significantly negatively correlated with tumor mutation burden (TMB) in both groups, and TMB combined with the m6A score could better predict the prognosis of patients and the effect of immunotherapy. This conclusion regarding the correlation between thyroid cancer and TMB is consistent with that of other studies, which also confirms the predictive effect of the m6A score. CTLA-4 and PD-1 are the most effective T cell immune checkpoint molecules that play a negative immunoregulatory role. A variety of ICIs have been approved for marketing for immunotherapy of clinical tumors, and immunotherapy for thyroid cancer has entered the clinical research stage. In this study, we explored the correlation of PD-L1 and CTLA-4 with m6A scores and confirmed that there were significant differences in the expression of the two checkpoints between the two m6A score groups. Therefore, this study shows that the m6A modification mode may have an impact on immune cell infiltration and the immunotherapy of thyroid cancer. Furthermore, through specific analysis of the prognosis, clinical characteristics, and TNM staging of patients with thyroid cancer, the m6A score was found to have a good prognostic significance in different subgroups. These results demonstrate the potential of the m6A score as an independent prognostic marker for thyroid cancer.

In this study, we reviewed and sorted the catalog of 23 m6A regulatory factors and included a series of newly identified m6A regulatory factors to optimize the accuracy of the m6A modification mode. Owing to the lack of appropriate ICI-based thyroid cancer datasets, we hope to further verify and improve the effect of m6A scores in combination with different immunotherapy schemes for other malignant tumors. A limitation of this study is that the m6A modification mode and m6A score were determined using retrospective data, and no prospective cohort study of patients with thyroid cancer undergoing immunotherapy is available to verify the results of this study. In addition, not all patients with lower m6A scores can benefit from ICIs treatment; therefore, more clinicopathological data need to be included in the prediction model to improve the accuracy of evaluation.

## CONCLUSION

In conclusion, this study demonstrated that m6A modification plays an important role in the tumorigenesis of thyroid cancer based on a large cohort. The m6A score can accurately predict the prognosis and clinical characteristics of patients with thyroid cancer, providing new insights and directions for exploring the potential pathogenesis of thyroid cancer and determining new targets for patient treatment.

## METHODS

### Data acquisition

We searched for public gene expression data and complete clinical annotations in The Cancer Genome Atlas (TCGA; https://cancergenome.nih.gov/) database. Patients without survival information or incomplete clinical data were excluded from further evaluation. In this study, 506 patients with thyroid cancer were collected from TCGA database for further analysis. Table 3 showed the clinicopathological characteristics of TC patients. For those from Affymetrix^®^, we downloaded the original “CEL” file and used affy and simplified software packages to perform background adjustment and quantile standardization using a robust multi-array averaging method. For microarray data from other platforms, the standardized matrix file was download directly. For the data-set from TCGA, the RNA sequencing data (FPKM value) of gene expression were downloaded from Genomic Data Commons (GDC, https://portal.gdc.cancer.gov/) using the R software package TCGAbiolinks, and then the FPKM value was converted into a transcript of one thousand base million (TPM) value. The “ComBat” algorithm of sva software package is used to correct the batch effect caused by non-biotechnology deviation. Somatic mutation data were obtained from TCGA database and analyzed using copy number variation (CNV). Immunohistochemical images of thyroid cancer were obtained from The Human Protein Atlas database (https://www.proteinatlas.org/). R software (version 4.0.3) and the R Bioconductor software package were used to analyze the data. The data used in this study conformed to the requirements of the official data published by TCGA and are publicly available.

**Table 3:** Clinicopathological characteristics of TC patients.

**Clinicopathological parameters**	**Total (*n* = 506) (%)**
Age	
Median (IQR)	47 (35–58)
Range (Min, Max)	15–89
<65	430 (85.0%)
≥65	76 (15.0%)
Gender	
Male	136 (26.9%)
Female	370 (73.1%)
Clinical T-stage	
T1	144 (28.5%)
T2	167 (33.0%)
T3	170 (33.6%)
T4	23 (4.5%)
Unknow	2 (0.4%)
Clinical N-stage	
N0	230 (45.4%)
N1	226 (44.7%)
Unknow	50 (9.9%)
Clinical M-stage	
M0	283 (55.9%)
M1	9 (1.8%)
Unknow	214 (42.3%)
TNM stage	
I	285 (56.3%)
II	52 (10.3%)
III	112 (22.1%)
IV	55 (10.9%)
Unknow	2 (0.4%)

### Expression analysis of m6A regulatory factor

The interaction information of 23 m6A regulatory factors was obtained from String Database (https://string-db.org), and the protein-protein interaction (PPI) network was constructed according to their expression relationship. The cut off standard was 0.7 interaction score (high confidence). The mRNA levels of 23 m6A regulators were analyzed using TPM data obtained from TCGA database. These 23 regulatory factors include eight encoder writers (METTL3, METTL14, METTL16, WTAP, KIAA1429/VIRMA, ZC3H13, RBM15, and RBM15B), 13 reader readers (YTHDC1, YTHDC2, YTHDF1, YTHDF2, YTHDF3, HNRNPA2B1, HNRNPC, IGFBP1, IGFBP2, IGFBP3, FMR1, LRPPRC, and RBMX) and two code cancellers erasers (ALKBH5 and FTO). The difference in expression between the normal group and the tumor group was detected using the R package limma (*P* < 0.05), with statistical significance.

### Survival and correlation analysis

The survival data of patients with thyroid cancer were obtained from their clinical data. Survival analysis was performed using R packet limma, survival, and surviviner. The survival curve was tested using Kaplan-Meier and log-rank tests. Pearson or Spearman correlation analysis was used for correlation analysis. R packet corrplot was used to draw the correlation heatmap, and R packet igrap, psych, reseape2, and RColorBrewer were used to draw the prognosis network map.

### m6A cluster

Based on the expression of 23 m6A molecules, unsupervised cluster analysis was used to identify different m6A modification patterns and classify the patients for analysis. All samples were divided into 2–9 to groups in turn. The most appropriate m6A clustering method was selected based on high clustering consistency, low coefficient of variation and lack of significant increase in the consistent cumulative distribution function (CDF) curve. The number and stability of clusters were determined using the consistent clustering algorithm. We use the R package ConsenseClusterPlus to perform the above steps and repeated them 1000 times to ensure the stability of the classification. To judge the fitness of classification, we drew the principal component analysis (PCA) diagram of m6A typing using R packet limma and ggplot2. Chi square test or Fisher exact test was used to analyze the clinicopathological characteristics and clustering, and the R package pheatmap was used to draw the heatmap. The Kaplan-Meier method was used to analyze the survival curve of the groups, and the log-rank test was used to determine the significance of the differences (*P* < 0.05). The overlapping differentially expressed genes (DEGs) among m6A groups were analyzed using Bayesian statistics of the R packet limma, and the *P* value was adjusted according to the error detection rate (FDR), with *P* < 0.001 as the screening standard. The R package clusterProfiler was used for GO enrichment analysis to explore the potential biological functions and pathways, and the potential biological functions and pathways (*p* < 0.05) were screened.

### Gene cluster

Single factor Cox regression analysis was carried out for DEGs with R-pack limma and survival, and prognosis related DEGs with *P* < 0.05 were screened. R package ConsensusClusterPlus was used to classify patients and identify gene clusters according to the expression of DEGs related to prognosis. All samples were divided into 2–9 groups in turn, and the most appropriate gene cluster typing method was selected. Chi square test or Fisher exact test was used to analyze the clinicopathological characteristics and clustering, and the R package pheatmap was used to draw the heat map. Survival analysis of gene clusters was carried out with the R package survival and surviviner, *P* < 0.05 was considered statistically significant. The expression of 23 m6A genes in different gene clusters was analyzed using the R packages limma, reshape2, and ggpubr.

### m6A score

To quantify the m6A modification pattern of a single tumor, we built a scoring system to evaluate the m6A modification pattern of TC patients, namely, the m6A score. We used a univariate Cox regression model to extract the DEGs with significant prognosis for principal component analysis and selected principal component 1 and principal component 2 as characteristic scores. The m6A score was defined as m6AScore = Σ (PC1i + PC2i), where i is the expression of m6A phenotype-related genes. Survival analysis was conducted with R package survival and surviviner to evaluate the prognostic value of the m6A score, and patients were divided into high- and low-rating groups according to their scores. The relationship between m6A typing, genotyping, m6A scoring group, and survival rate was studied using the R packages ggalleuvial, ggplot2, and dplyr, and the Alluvial diagram was drawn to show the results. The difference in m6A score in m6A classification and genotyping was compared using the R packages limma and ggpubr, *P* < 0.05 was considered statistically significant.

### Immunophenoscore (IPS) analysis

The Cancer Immune Atlas database (TCIA; https://tcia.at/home). The next generation sequencing (NGS) data of more than 9500 tumor samples from 20 solid cancers were collected from the Cancer Genome Map (TCGA) and other databases, and the immune phenotype score (IPS) of tumor samples can be detected, which can predict the response of tumors to cytotoxic T lymphocyte antigen-4 (CTLA-4) and programmed cell death protein 1 (PD-1) blockers. We obtained 507 IPS of RC samples through the TCIA database, divided RC samples into high expression group and low expression group according to the median m6A score, and analyzed the relationship between m6A score and IPSs using the chi square test to further clarify the correlation between m6A score and immunotherapy response. Combined with the clinical data of patients, the survival curves of patients with different m6A scores under different clinical and pathological characteristics were drawn using R packet survival and surviviner.

### Statistical analysis

All statistical analyses were performed using the R software (version 4.0.3). Chi-square or Fisher’s exact test was used to classify variables, and Wilcoxon or Kruskal Wallis test was used to compare gene expression between different samples. Pearson or Spearman correlation analysis was used to evaluate the correlations between the two variables. We used a univariate Cox regression model to calculate the risk ratio (HR) of the related genes. Statistical significance was set at *P* < 0.05.

## Supplementary Materials

Supplementary Figures
