# Supplementary material for: Thyroid cancer risk prediction model using m6A RNA methylation regulators: integrated bioinformatics analysis and histological validation
Source: Aging (Albany NY). 2023 Feb 15;15(3):846–65. doi: 10.18632/aging.204525 (PMC9970309; doi:10.18632/aging.204525)
Supplement: Supplementary Figures [file aging-15-204525-s001.pdf]

SUPPLEMENTARY FIGURES

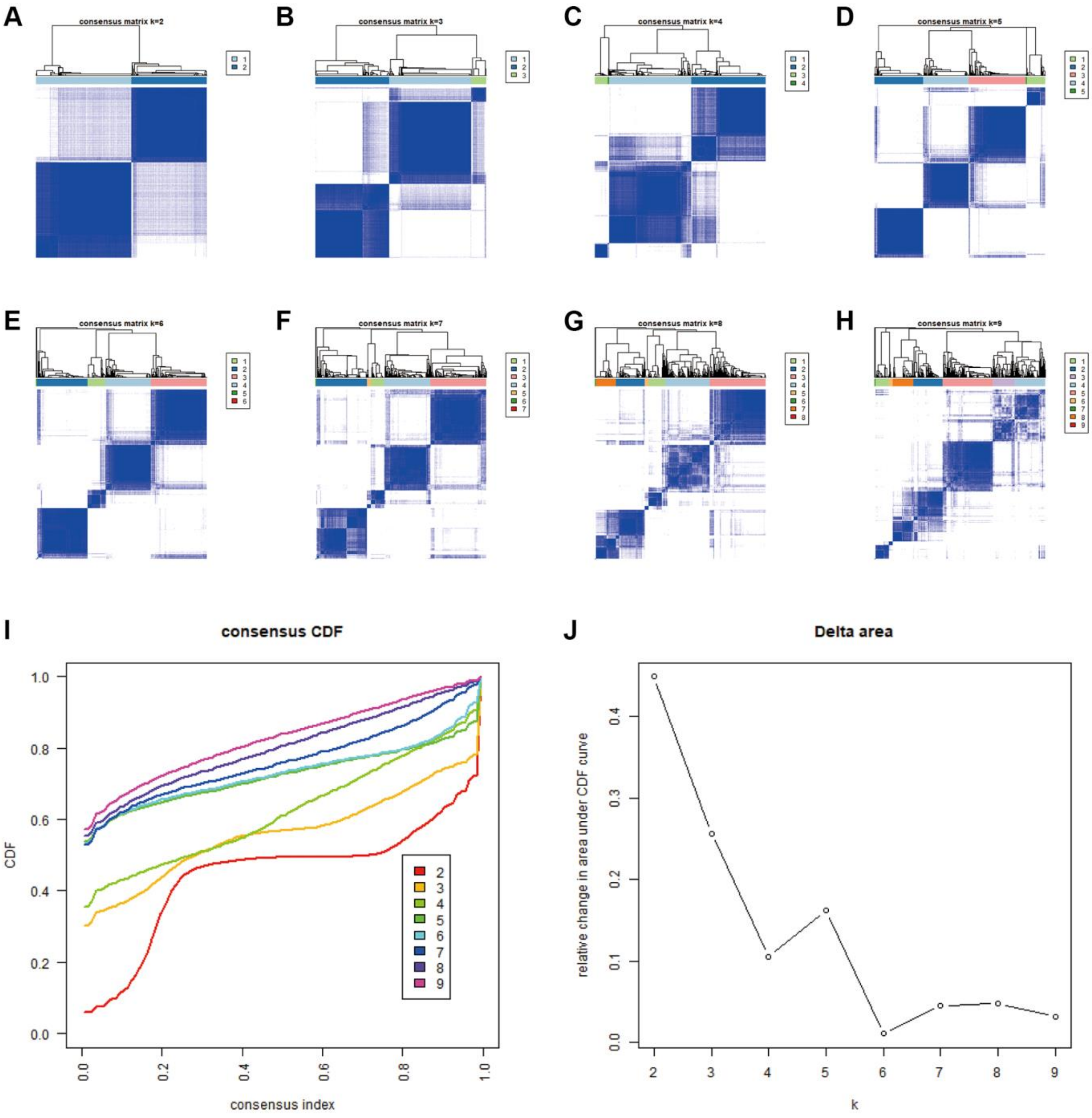

Supplementary Figure 1. Consensus clustering analysis of m6A modification clusters. (**A**–**H**) Consensus matrices of the patients with sarcoma for *k* = 2–9. (**I**) Cumulative distribution function (CDF) of consensus clustering analysis. (**J**) Relative change in area under the CDF curve of consensus clustering analysis.

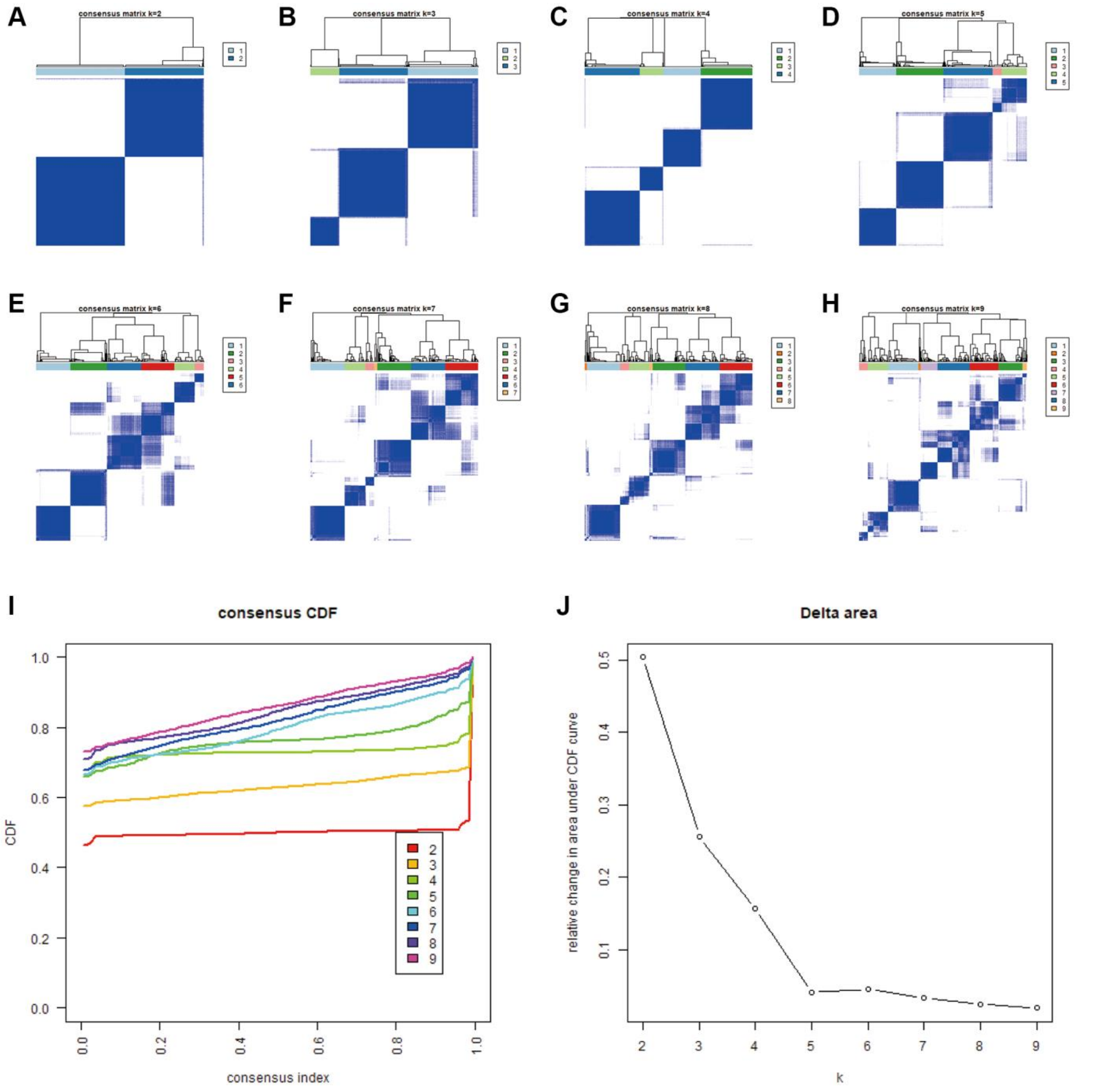

Supplementary Figure 2. Consensus clustering analysis of m6A gene clusters. (**A**–**H**) Consensus matrices of the patients with sarcoma for *k* = 2–9. (**I**) CDF of consensus clustering analysis. (**J**) Relative change in area under the CDF curve of consensus clustering analysis.
